# A Novel Model for Vulnerability Analysis through Enhanced Directed Graphs and Quantitative Metrics

**DOI:** 10.3390/s22062126

**Published:** 2022-03-09

**Authors:** Ángel Longueira-Romero, Rosa Iglesias, Jose Luis Flores, Iñaki Garitano

**Affiliations:** 1 Ikerlan Technology Research Centre, Basque Research and Technology Alliance (BRTA), 20500 Arrasate, Spain; riglesias@ikerlan.es (R.I.); jlflores@ikerlan.es (J.L.F.); 2 Department of Electronics and Computing, Mondragon Unibertsitatea, 20500 Mondragón, Spain; igaritano@mondragon.edu

**Keywords:** CPE, CVE, CVSS, CWE, CAPEC, directed graph, IACS, cybersecurity, vulnerability assessment, security metrics, IEC 62443, OpenPLC

## Abstract

The rapid evolution of industrial components, the paradigm of Industry 4.0, and the new connectivity features introduced by 5G technology all increase the likelihood of cybersecurity incidents. Such incidents are caused by the vulnerabilities present in these components. Designing a secure system is critical, but it is also complex, costly, and an extra factor to manage during the lifespan of the component. This paper presents a model to analyze the known vulnerabilities of industrial components over time. The proposed Extended Dependency Graph (EDG) model is based on two main elements: a directed graph representation of the internal structure of the component, and a set of quantitative metrics based on the Common Vulnerability Scoring System (CVSS). The EDG model can be applied throughout the entire lifespan of a device to track vulnerabilities, identify new requirements, root causes, and test cases. It also helps prioritize patching activities. The model was validated by application to the OpenPLC project. The results reveal that most of the vulnerabilities associated with OpenPLC were related to memory buffer operations and were concentrated in the *libssl* library. The model was able to determine new requirements and generate test cases from the analysis.

## 1. Introduction

Industrial components are the driving force of almost every industrial field, such as automotive, energy production, and transportation [1,2,3,4,5,6]. These types of components are rapidly evolving [7,8] and increasing in number [9]. This increase is related to several factors: (1) the reuse of open-source hardware and software, (2) new connectivity features, and (3) more complex systems.

Open-source hardware and software, and Commercial Off-The-Shelf (COTS) components are being integrated to speed up their development [10,11,12]. COTS are easy to use, but they can introduce vulnerabilities, creating potential entry points for attackers [13,14].

Industrial components are providing more advanced connectivity features, enabling new automation applications, services, and data exchange. This new connectivity, boosted by the fifth generation (5G) of wireless technology for cellular networks, will further open the window of exposure to any threat [6,9,15,16].

The complexity of industrial systems is also increasing with the integration of new trends, such as the Internet of Things (IoT) [16,17,18,19], cloud computing, Artificial Intelligence (AI) [19,20], and big data. The extensive use of these technologies further opens the windows for attackers [21,22,23,24,25,26]. Complexity is a critical aspect of industrial components design because it is closely related to the number of vulnerabilities [27,28].

This scenario points to security as a key aspect of industrial components. Moreover, numerous attacks have been reported targeting industrial enterprises across the globe since 2010 [29]. An exponential rise in such attacks is predicted for future years [30,31].

Although great efforts are being made to develop new and better ways to analyze vulnerabilities [32,33], to measure them (e.g., Common Vulnerabilities and Exposures (CVE) [34], Common Vulnerability Scoring System (CVSS) [35,36,37], or Common Weakness Enumeration (CWE) [38,39]), or to aggregate them [40], to the best of our knowledge, existing models do not cover the entire life cycle of industrial components. Performing a vulnerability analysis at a single point in time (e.g., during development or when a product has been released) is not enough for industrial components, and their long lifespan has to be considered [41,42]. Furthermore, both software and hardware should be considered, given the strong bonding between hardware and software in industrial components [43,44,45,46].

In the present paper, we propose an Extended Dependency Graph (EDG) model that performs continuous vulnerability assessment to determine the source and nature of vulnerabilities and enhance security throughout the entire life cycle of industrial components. The proposed model is built on a directed graph-based structure, and a set of metrics based on globally accepted security standards.

This paper is structured as follows: First, the related work is reviewed in Section 2. Then, the main pieces of the proposed model are defined in Section 3. Second, to demonstrate the potential of this proposal, the proposed model is applied to a real use case in Section 4. Finally, conclusions and future work of this research are described in Section 5.

## 2. Related Work

This section will review the current status of vulnerability assessment. This review aims to find similar approaches from the literature, including the current standard and metrics.

### 2.1. Vulnerability Analysis in Security Standards

Industry is currently making a significant effort to incorporate security aspects into the development of industrial components, which has led to a set of standards, such as the Common Criteria and ISA/IEC 62443. This review is focused on how these standards conduct vulnerability analysis, the use of metrics, their management of the life cycle of the device, the techniques that they propose, and the security evaluation of both software and hardware.

#### 2.1.1. ISA/IEC 62443

ISA/IEC 62443 constitutes a series of standards, technical reports, and related information that define the procedures and requirements for implementing electronically secure Industrial Automation and Control Systems (IACSs) [47]. As expressed by this standard, security risk management shall jointly and collaboratively be addressed by all the entities involved in the design, development, integration, and maintenance of the industrial and/or automation solution (including subsystems and components) to achieve the required security level [48].

This joint effort is reflected in the organization of the documents of the standard, which is divided into four parts:

1. Part 1—General: Provides background information such as security concepts, terminology, and metrics;
2. Part 2—Policies and procedures: Addresses the security and patch management policies and procedures;
3. Part 3—System: Provides system development requirements and guidance;
4. Part 4—Component: Provides product development and technical requirements, which are intended for product vendors.

The ISA/IEC 62443-4-1 technical document is divided into eight practices, which specify the secure product development life cycle requirements for both the development and the maintenance phases [49]. The “Practice 5—Security verification and validation testing” (SVV) section of this document specifies that a process shall be employed to identify and characterize potential security vulnerabilities in the product, including known and unknown vulnerabilities [50,51]. Two requirements in Practice 5 are in charge of the task of analyzing vulnerabilities, as follows:

- Requirement SVV-3. Vulnerability Testing [49]. This requirement states that a process shall be employed to perform tests that focus on identifying and characterizing potential and known security vulnerabilities in the product (i.e., fuzz testing, attack surface analysis, black box known vulnerability scanning, software composition analysis, and dynamic runtime resource management testing).
- Requirement SVV-4. Penetration Testing [49]. This requirement states that a process shall be employed to identify and characterize security-related issues via tests that focus on discovering and exploiting security vulnerabilities in the product (i.e., penetration testing).

Although the ISA/IEC 62443-4-1 document considers the possibility of analyzing and characterizing the vulnerabilities of an industrial component, it does not propose a technique to perform this task but instead refers to other standards for vulnerability handling processes [52]. In addition, it does not indicate how the data obtained from the analysis should be interpreted, and it does not define metrics or reference values for the current state of compliance with the requirement. Finally, it does not take into account neither the dependencies among the assets of the industrial component (dependency trees) or their evolution of the number of vulnerabilities over time.

#### 2.1.2. Common Criteria

The Common Criteria (CC) for Information Technology Security Evaluation (ISO/IEC 15408) is an international standard that has a long tradition in computer security certification [53]. CC is a framework that provides assurance that the processes of specification, implementation, and evaluation of a computer security product have been conducted in a rigorous, standard, and repeatable manner at a level that is commensurate with the target environment for use.

To describe the rigor and depth of an evaluation, the CC defines seven Evaluation Assurance Levels (EALs) on an increasing scale [53], from EAL1 (the most basic) to EAL7 (the most stringent security level). It is important to notice that the EAL levels do not measure security itself. Instead, emphasis is given to functional testing, confirming the overall security architecture and design, and performing some testing techniques (depending on the EAL to be achieved).

The CC defines five tasks in the vulnerability assessment class, which manage the deepness of the vulnerability assessment. The higher the EAL to be achieved, the greater the number of tasks in the list to be performed [54]:

1. Vulnerability survey,
2. Vulnerability analysis,
3. Focused vulnerability analysis,
4. Methodical vulnerability analysis, and
5. Advanced methodical vulnerability analysis.

Every task checks for the presence of publicly known vulnerabilities. Penetration testing is also performed. The main difference among the five levels of vulnerability analysis described here is the deepness of the analysis of known vulnerabilities and the penetration testing.

The CC scheme defines the general activities, but it does not specify how to perform them, therefore no technique for analyzing vulnerabilities is proposed. The evaluator decides the most appropriate techniques for each test in each scenario and for each device, which adds a large degree of subjectivity to the evaluation. Furthermore, dependencies among vulnerabilities and assets are not considered in the analysis. Moreover, the CC does not define a procedure to manage the life cycle of the device. In other words, when updated, the whole device has to be reevaluated [55,56,57,58]. Finally, although the usage of metrics is encouraged by the CC, it does not propose any explicitly defined metric to be used during the evaluation.

### 2.2. Vulnerability Analysis Methodologies

Vulnerability analysis is a key step towards the security evaluation of a device. Consequently, many research efforts have been focused on solving this issue. In this subsection, the most relevant works related to vulnerability analysis are reviewed.

Homer et al. [59] present a quantitative model for computer networks that objectively measures the likelihood of a vulnerability. Attack graphs and individual vulnerability metrics, such as CVSS and probabilistic reasoning are applied to produce a sound risk measurement. However, the main drawback is that their work is only applicable to computer networks. Although they propose new metrics based on the CVSS for probabilistic calculations, they do not integrate standards such as CAPEC to enhance their approach centered on possible attacks and privilege escalation. They also fail to establish a relationship among existing vulnerabilities, and they fail to obtain the source problem causing each vulnerability.

Zhang et al. [60,61] developed a quantitative model that can be used to aggregate vulnerability metrics in an enterprise network based on attack graphs. Their model measures the likelihood that breaches can occur within a given network configuration, taking into consideration the effects of all possible interplays between vulnerabilities. This research is centered on computer networks, using attack graphs. Although the proposed model is capable of managing shared dependencies and cycles, only CVSS-related metrics are used. Moreover, this model assumes that the attacker knows all of the information in the generated attack graphs. Finally, the method that they proposed for the aggregation of metrics is not valid for vulnerability analysis, because the dependency between vulnerabilities reflected in attacks graphs are is not trivially obtained.

George et al. [30] propose a graph-based model to address the security issues in Industrial IoT (IIoT) networks. Their model is useful because it represents the relationships among entities and their vulnerabilities, serving as a security framework for the risk assessment of the network. Risk mitigation strategies are also proposed. Finally, the authors discuss a method to identify the strongly connected vulnerabilities. However, the main drawback of this work is that each node of the generated attack graph represents a vulnerability instead of representing a device or an asset of that device. This leads to a loss of information in the analysis because there is no way to know which vulnerability belongs to which device. Moreover, these methods need to know the relationships among present vulnerabilities in the devices. This information is not trivially obtained, and a human in the loop is needed. The proposals of [62,63] follow a similar graph-based approach to study the effects of cascade failures in the power grid and a subway network.

Poolsappasit et al. [64] propose a risk management framework using Bayesian networks that enables a system administrator to quantify the chances of network compromise at various levels. The authors are able to model attacks on the network, and also to integrate standardized information of the vulnerabilities involved, such as their CVSS score. Although their proposed model lends itself to dynamic analysis during the deployed phase of the network, these results can only be applied to computer networks where the relationship among the existing vulnerabilities is known. Meanwhile, the prior probabilities that are used in the model are assigned by network administrators, and hence are subjective. The proposed model also has some issues related to scalability.

Muñoz-González et al. [65] propose the use of efficient algorithms to make an exact inference in Bayesian attack graphs, which enables static and dynamic network risk assessments. This model is able to compute the likelihood of a vulnerability and can be extended to include zero-day vulnerabilities, attacker’s capabilities, or dependencies between vulnerability types. Although this model is centered on studying possible attacks, it fails to integrate standards (such as CAPEC) that are related to attack patterns. Moreover, the generated graphs are focused on privilege escalation, trust, and users, rather than including information about vulnerabilities and the analyzed device.

Liu et al. [66] carry out a detailed assessment of vulnerabilities in IoT-based critical infrastructures from the perspectives of applications, networking, operating systems, software, firmware, and hardware. They highlight the three key critical infrastructure IoT-based cyber-physical systems (i.e., smart transportation, smart manufacturing, and smart grid). They also provide a broad collection of attack examples upon each of the key applications. Finally, the authors provide a set of best practices and address the necessary steps to enact countermeasures for any generic IoT-based critical infrastructure system. Nevertheless, their proposal is focused on attacks and countermeasures, and it leaves aside the inner analysis of the targets. Continuous evaluation over time is not considered in this proposal, and no enhancements of the development process are generated. On the other hand, Pascale et al. [67] proposed the analysis in both spatial and temporal dimensions for intrusion detection.

Hu et al. [68] propose a network security risk assessment method that is based on the Improved Hidden Markov Model (I-HMM). The proposed model reflects the security risk status in a timely and intuitive manner, and it detects the degree of risk that different hosts pose to the network. Although this is a promising approach, it is centered on computer networks and is at a higher abstraction level. No countermeasure or enhancement in the development process is proposed or generated.

Zografopoulos et al. [13] provide a comprehensive overview of the Cyber-Physical System (CPS) security landscape, with an emphasis on Cyber-Physical Energy Systems (CPES). Specifically, they demonstrate a threat modeling methodology to accurately represent the CPS elements, their interdependencies, as well as the possible attack entry points and system vulnerabilities. They present a CPS framework that is designed to delineate the hardware, software, and modeling resources that are required to simulate the CPS. They also construct high-fidelity models that can be used to evaluate the system’s performance under adverse scenarios. The performance of the system is assessed using scenario-specific metrics. Meanwhile, risk assessment enables system vulnerability prioritization, while factoring in the impact on the system’s operation. Although this research work is comprehensive, it is focused on enhancing the existing adversary and attack modeling techniques of CPSs of the energy industry. Moreover, their model does not integrate the internal structure of the target of evaluation, and it does not take both software and hardware into account for the evaluation. Continuous evaluation over time is not considered. Finally, they do not propose countermeasures or any kind of mechanism to enhance the security or the development of the CPSs.

Most of the works reviewed here are more focused on modeling threats and attacks, instead of using their results to propose enhancements during other steps in the life cycle of CPS (e.g., development, and maintenance). It is worth noting that they are still more focused on software evaluation, while hardware is usually neglected in their proposals.

As shown in this review, most of the research has adopted dependency trees, attack graphs, or directed graphs as the main tool to manage and assess vulnerabilities in computer networks. Graphs are an efficient technique to represent the relationships between entities, and they can also effectively encode the vulnerability relations in the network. Furthermore, the analysis of the graph can reveal the security-relevant properties of the network. For fixed infrastructure networks, graphical representations, such as attack graphs, are developed to represent the possible attack paths by exploiting the vulnerability relationships. For these reasons, vulnerability analysis techniques based on directed graphs are frequently found in the literature [69]. However, despite their potential, these analysis techniques have been relegated to vulnerability analysis in computer networks. Graph-based analysis has rarely been applied to industrial components.

### 2.3. Security Metrics

Standards of measurement and metrics are a powerful tool to manage security and for making decisions [70,71,72]. If carefully designed and chosen, metrics can provide a quantitative, repeatable, and reproducible value. This value is selected to be related to the property of interest of the systems under test (e.g., number and distribution of vulnerabilities). The use of metrics enables results to be compared over time, and among different devices. In addition, they can also be used to systematically improve the security level of a system or to predict this security level at a future point in time.

Although the capabilities of metrics have been demonstrated, they are not free of drawbacks. In our previous research work [72], we performed a systematic review of the literature and standards. To detect possible gaps, our objective was to find which types of metrics have been proposed and in which fields have been applied. This research work concludes that, in general, standards encourage the use of metrics, but they do not usually propose any specific set of metrics. If metrics are proposed, then they are conceived to be applied at a higher level (i.e., organization level), and then cannot be applied to industrial components. This type of metric is usually related to measuring the return on security investment, security budget allocation, and reviewing security-related documentation.

Our previous results also highlight that scientific papers have focused their efforts on software-related metrics: 77.5% of the analyzed metrics were exclusively applicable to software (e.g., lines of code, number of functions, and so on), whereas only 0.6% were related exclusively to hardware (e.g., side-channel vulnerability factor metric). In addition, 14.8% of them could be applied to both software and hardware (e.g., the historically exploited vulnerability metric that measures the number of vulnerabilities exploited in the past), and the remaining 7.1% are focused on other aspects, such as user usability. This shows that there is a clear lack of hardware security metrics in the literature, and the main contributions are centered on software security.

Other research works also reveal common problems across security metrics [73,74]:

- Hardly any security metric has a solid theoretical foundation or empirical evidence in support of the claimed correlation.
- Many security metrics lack an adequate description of the scale, unit, and reference values to compare and interpret the results.
- Only a few implementations or programs were available to test these security metrics and only one of the analyzed papers performed some kind of benchmarking or comparison with similar metrics.
- The information provided in the analyzed papers is insufficient to understand whether the proposed metrics are applicable in a given context, or how to use them.

Under this scenario, it seems reasonable that future research should be focused on the development of a convincing theoretical foundation, empirical evaluation, and systematic improvement of existing approaches, in an attempt to solve the lack of widely accepted solutions. In this research work, metrics constitute a key element. They are developed to analyze the distribution of vulnerabilities and to track their evolution over time.

## 3. Proposed Approach

In this research work, we propose an EDG model for the continuous assessment of vulnerabilities over time in industrial components. The proposed model is intended to:

- Identify the root causes and nature of vulnerabilities, which will enable the extraction of new requirements and test cases.
- Support the prioritization of patching.
- Track vulnerabilities during the whole lifespan of industrial components.
- Support the development and maintenance of industrial components.

To accomplish this task, the proposed model comprises two basic elements: (1) the model itself, which is capable of representing the internal structure of the system under test; (2) a set of metrics, which allow conclusions to be drawn about the origin, distribution, and severity of vulnerabilities. Both the model and metrics are very flexible and exhibit some properties that make them suitable for industrial components, and can also be applied to enhance the ISA/IEC 62443 standard.

The content in this section is distributed into four sections, namely:

1. Model: The proposed model is explained, together with the systems in which it can be applied and the algorithms that are used to build it.
2. Metrics: Metrics are a great tool to measure the state of the system and to track its evolution. The proposed metrics and their usage are described in this section.
3. Properties: The main features of the proposed model and metrics (e.g., granularity of the analysis, analysis over time, and patching policy prioritization support) are described in detail.
4. Applicability: Even though the reviewed standards exhibit some gaps, the proposed model aims to serve as the first step towards generating a set of tools to perform a vulnerability analysis in a reliable and continuous way. This last section will discuss the requirements of the ISA/IEC 62443-4-1 that can be enhanced using our model.

### 3.1. Description of the Model

The proposed model is based on directed graphs. It requires knowledge of the internal structure of the device to be evaluated (i.e., the assets, both hardware and software, that comprise it and the relationships between them). This section defines the most basic elements that make up the model, the algorithms to build it for any given system, and its graphical representation.

**Definition** **1.** *A System Under Test (SUT) (following the denomination in the ISA/IEC 62443 standard [47], the SUT may be an industrial component, a part of an industrial component, a set of industrial components, a unique technology that may never be made into a product, or a combination of these) is now represented by an Extended Dependency Graph (EDG) model $G=\left(\langle A,V\rangle ,E\right)$ that is based on directed graphs, where A and V represent the nodes of the graphs, and E represents its edges or dependencies:*

- *$A=\{a_{1},\ldots ,a_{n}\}$ represents the set of assets in which the SUT can be decomposed, where n is the total number of obtained assets. An asset a is any component of the SUT that supports information-related activities and includes both hardware and software [75,76,77]. Each asset is characterized by its corresponding Common Platform Enumeration (CPE) [78,79,80] identifier, while its weaknesses are characterized by the corresponding CWE identifier. In the EDG model, the assets are represented by three types of nodes in the directed graphs (i.e., root nodes, asset nodes, and cluster).*
- *$V=\{v_{1},\ldots ,v_{q}\}$ represents the set of known vulnerabilities that are present in each asset of A, where q is the total number of vulnerabilities. They are characterized by the corresponding CVE and CVSS values. In the EDG model, vulnerabilities are represented using two types of nodes in the directed graphs (i.e., known vulnerability nodes and clusters).*
- *$E=\{e_{ij}|\forall i,j\in \left\{1,\ldots ,n+q\right\}\;such\;that\;i\neq j\}$ represents the set of edges or dependencies among the assets, and between assets and vulnerabilities. $e_{ij}$ indicates that a dependency relation is established from asset $a_{i}$ to asset $a_{j}$. Dependencies are represented using two different types of edges in the EDG (i.e., normal dependency and deprecated asset/updated vulnerability edges).*

In other words, the EDG model can represent a system, from its assets to its vulnerabilities, and its dependencies as a directed graph. Assets and vulnerabilities are represented as nodes, whose dependencies are represented as arcs in the graph. The information in the EDG is further enhanced by introducing metrics.

**Figure 1:**
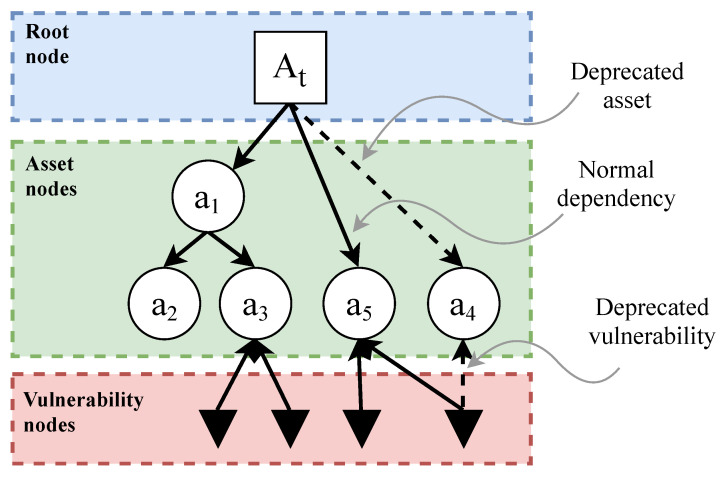
Basic elements of an EDG. Note that clusters are not displayed in this figure. For clusters, see Figure 2. For metric definitions, see Section 3.2.

The EDG model of a given SUT will include four types of node and two types of dependency. The graphical representation for each element is shown in Table 1. Figure 1 shows an example of a simple EDG and its basic elements. All of the elements that make up an EDG will be explained in more detail below:

#### 3.1.1. Types of Node

The EDG model uses four types of nodes:

- Root nodes represent the SUT,
- Asset nodes represent each one of the assets of the SUT,
- Known vulnerability nodes represent the vulnerabilities in the SUT, and
- Clusters summarize the information in a subgraph.

Root nodes (collectively, set $G_{R}$) are a special type of node that represents the whole SUT. Any EDG starts in a root node and each EDG will only have one single root node, with an associated timestamp (t) that indicates when the last check for changes was done. This timestamp is formatted following the structure defined in the ISO 8601 standard for date and time [81].

Asset nodes (collectively, set $G_{A}$) represent the assets that comprise the SUT. The EDG model does not impose any restrictions on the minimum number of assets that the graph must have. However, the SUT can be better monitored over time when there is a higher number of assets. Moreover, the results and conclusions obtained will be much more accurate. Nevertheless, each EDG will have as many asset nodes as necessary, and the decomposition of assets can go as far and to as low-level as needed.

Each asset node node will be characterized by the following set of values:

- $CPE_{current}$: Current value for the CPE. This points to the current version of the asset it refers to.
- $CPE_{previous}$: Value of the CPE that identifies the previous version of this asset. This will be used by the model to trace back all the versions of the same asset over time, from the current version to the very first version.
- $CWE_{a_{i}}\left(t\right)$: Set of all the weaknesses that are related to the vulnerabilities present in the asset. The content of this list can vary depending on the version of the asset.

Figure 3 illustrates how the tracking of the versions of an asset using CPE works. On the one hand, version $a_{i}$ is the current version of asset *a*. It contains its current CPE value and the CPE of its previous version. On the other hand, $a_{2}$ and $a_{1}$ are previous versions of asset *a*. The last value of $a_{1}$ points to a null value. This indicates that it is the last value in the chain, and therefore the very first version of the asset *a*.

Known vulnerability nodes (collectively, set $G_{V}$) represent a known vulnerability present in the asset that it relates to. Each asset will have a known vulnerability node for each known vulnerability belonging to that asset. Assets alone cannot tell how severe or dangerous the vulnerabilities might be, so unique characterization of vulnerabilities is crucial [30].

To identify each known vulnerability node, each will be characterized by the following set of features (formally defined in Section 3.2:

- $CVE_{a_{i}}\left(t\right)$: This serves as the identifier of a vulnerability of asset $a_{i}$.
- $CVSS_{v_{i}}\left(t\right)$: This metric assigns a numeric value to the severity of vulnerability $v_{i}$. Each CVE has a corresponding CVSS value.
- $CAPEC_{w_{i}}\left(t\right)$: Each vulnerability (CVE) is a materialization of a weakness (CWE) $w_{i}$ that can be exploited using a concrete attack pattern. In many cases, each CWE has more than one Common Attack Pattern Enumeration and Classification (CAPEC) [82,83] associated. Consequently, this field is a set that contains all the possible attack patterns that can exploit the vulnerability that is being analyzed.

**Figure 2:**
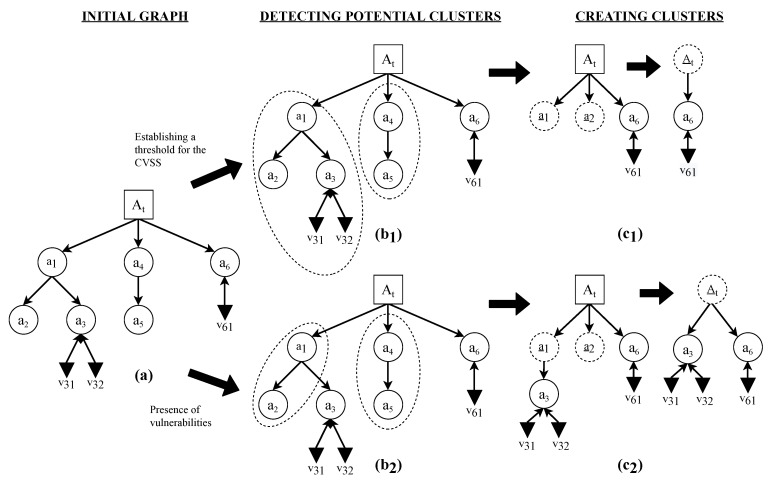
Creating clusters. Application of the two proposed criteria to the creation of clusters to simplify the graph, where (**a**) represents the initial EDG: (1) Establishing a threshold to select which vulnerability stays outside the cluster (upper side). In step (**b_1_**), potential clusters are detected according to the established threshold, while in (**c_1_**) the final EDG with the generated clusters is shown. The severity value (CVSS) for v_31_ and v_32_ is supposed to be lower than the establish threshold. (2) Choosing the absence of vulnerabilities as the criterion to create clusters (lower side). In step (**b_2_**), nodes with no vulnerability are detected. In (**c_2_**), the final EDG with the generated clusters is shown.

**Figure 3:**
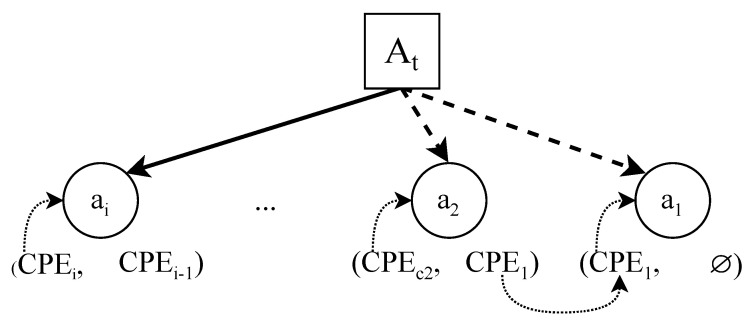
Tracking dependencies between the previous and current CPE values for asset *a*.

Clusters (collectively, set $G_{S}$) are a special type of node that summarizes and simplifies the information contained in a subgraph in an EDG. Figure 2 shows how the clusters work.

To identify each cluster, and to be able to recover the information that they summarize, each is characterized by the data that define each of the elements that they contain: $\left\{CPE_{previous},CPE_{current},CWE_{a_{i}}\left(t\right)\right\}$, $\left(CVE_{a_{i}}\left(t\right),CVSS_{v_{i}}\left(t\right),\left\{CAPEC_{w_{i}}\left(t\right)\right\}\right)$, and their dependencies.

Two types of criteria can be used to create clusters and to simplify the obtained graph Figure 2:

1. Absence of vulnerabilities: Using this criterion, clusters will group all nodes that contain no associated vulnerabilities.
2. CVSS score below a certain threshold: With this criterion, a threshold for the CVSS scores will be chosen. Nodes whose CVSS score is less than the defined threshold will be grouped into a cluster.

#### 3.1.2. Types of Edge

In the EDG model, edges play a key role in representing dependencies. Two types of edge can be identified:

- Normal dependencies relate two assets, or an asset and a vulnerability. They represent that the destination element depends on the source element. Collectively, they are known as set $G_{D}$.
- Deprecated asset or patched vulnerability dependencies indicate when an asset or a vulnerability is updated or patched. They represent that the destination element used to depend on the source element. Collectively, they are known as set $G_{U}$.

The possibility of representing old dependencies brings the opportunity to reflect the evolution of the SUT over time. When a new version of an asset is released, or a vulnerability is patched, the model will be updated. Their dependencies will change from a normal dependency to a deprecated asset or vulnerability dependency to reflect that change.

#### 3.1.3. Conditions of Application of EDGs

The EDG model is applicable to SUTs that meet the following set of criteria:

- Software and hardware composition: In our approach, the model is created by means of a white-box analysis. The absence of or impossibility to perform a white-box analysis limits the ability to create an accurate model. Some knowledge about the internal structure and code is expected. This information is usually only known by the manufacturer of the component unless the component is publicly available or open-source. It should be also possible to decompose the SUT into simpler assets to generate a relevant EDG.
- Existence of publicly known vulnerabilities: The EDG model focuses on known vulnerabilities. This is not critical because many industrial components use commercial or open-source elements. The SUT must be composed of assets for which public information is available. If the majority of SUT assets are proprietary, or the SUT is an ad hoc development that is never exposed, then the generated EDG will not evolve. Therefore, the analysis will not be relevant.

#### 3.1.4. Steps to Build the Model

This section explains the process and algorithms that were used to build the corresponding EDG of a given SUT. The main scenarios that can be found are also described.

**Figure 4:**
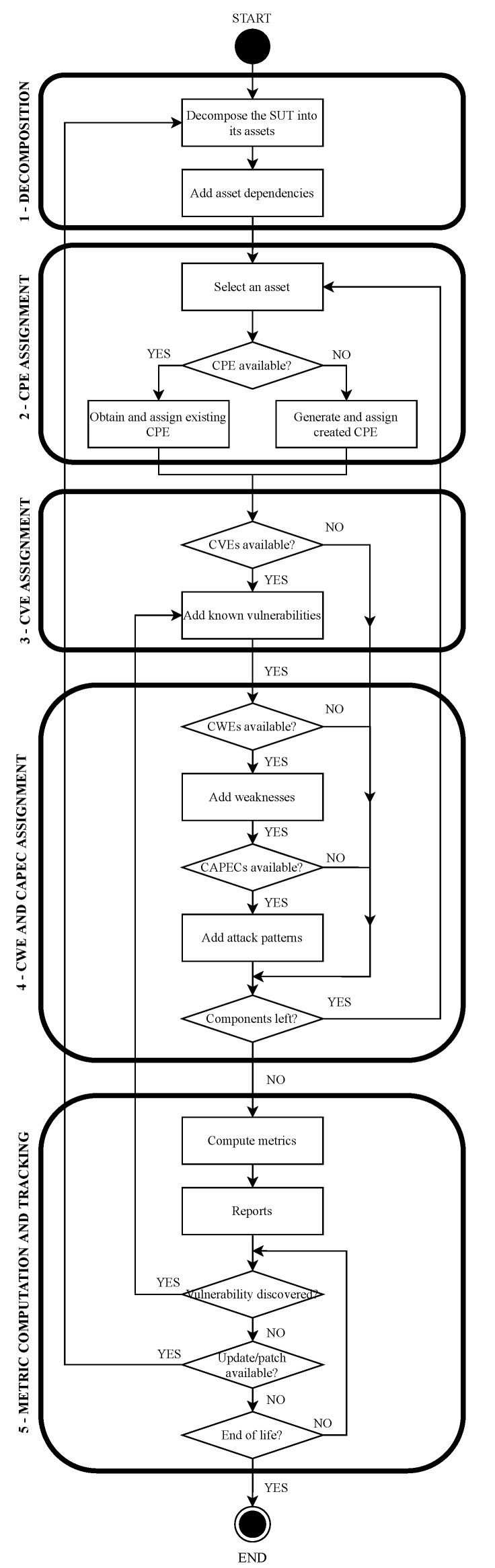
Algorithm to generate the initial EDG of a given SUT.

**Figure 5:**
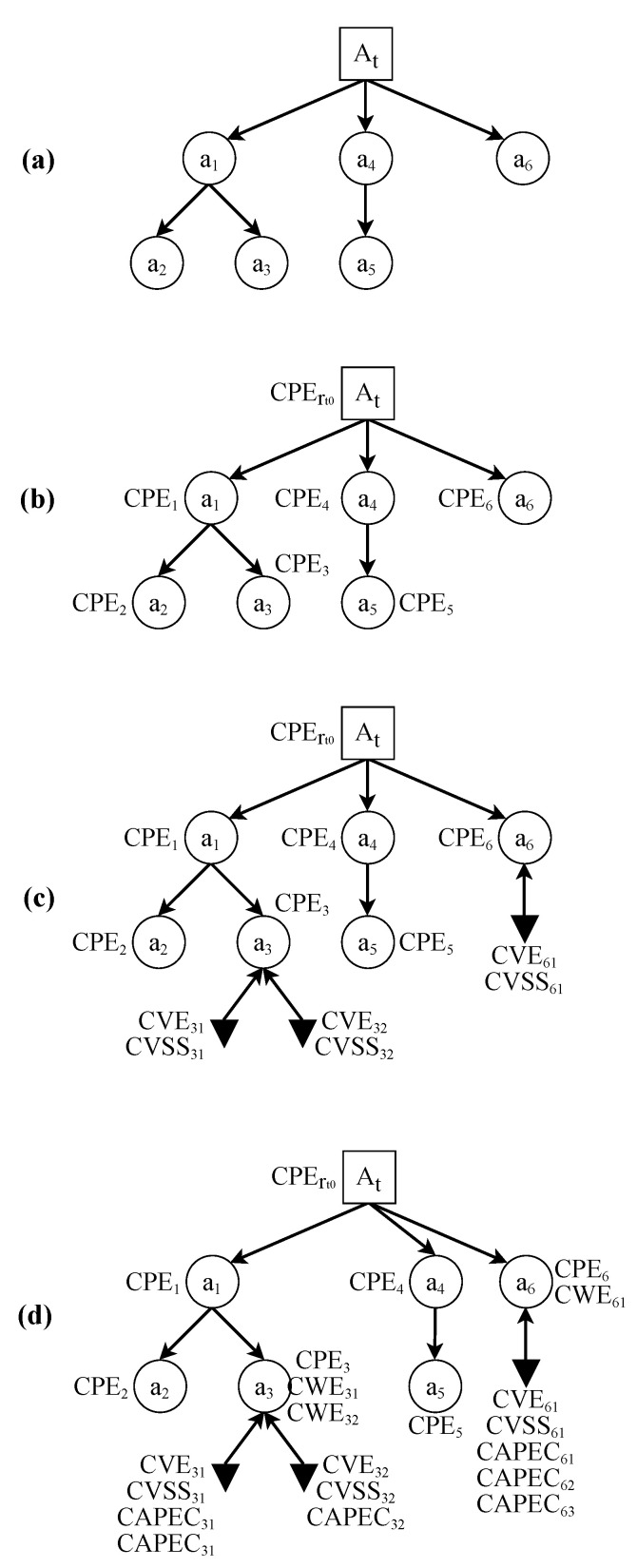
Example of the process of building the EDG model of a given SUT *A*. (**a**) Decompose of the SUT into assets. (**b**) Assign a CPE to each asset. (**c**) Add known vulnerabilities. (**d**) Add weaknesses and attack patterns.

Before extracting useful information about the SUT, the directed graph associated with the SUT has to be built. This comprises several steps, which are described in the following paragraphs (see the flowchart in Figure 4 and Figure 5):

Step 1—Decompose the SUT into assets. For the model to work properly, it relies on the SUT being able to be decomposed into assets. With this in mind, the first step involves obtaining the assets of the SUT, either software or hardware. In the CC, this process is called modular decomposition of the SUT [53]. Ideally, every asset should be represented in the decomposition process, but this is not compulsory for the model to work properly. Each one of the assets obtained in this step will be represented as an asset node. In this step, the dependencies among the obtained assets are also added as normal dependencies.

Step 2—Assign a CPE to each asset. Once the assets and their dependencies have been identified, the next task is to assign the corresponding CPE identifier to each asset. If there is no publicly available information of a certain asset, and therefore, it does not have a CPE identifier, then it is always possible to generate one using the fields described in the CPE naming specification documents [79] for internal use in the model.

Step 3—Add known vulnerabilities to the assets. In this step, the vulnerabilities ($CVE_{a_{i}}\left(t\right)$) of each asset are set. This is done by consulting public databases of known vulnerabilities [34,84] looking for existing vulnerabilities for each asset. When a vulnerability is found, it is added to the model of the SUT, including its dependencies. If there were no known vulnerabilities in an asset, then the asset would become the last leaf of its branch. In this step, the corresponding value of the CVSS of each vulnerability is also added to the model.

Step 4—Assign to each asset its weaknesses and possible CAPECs. After the vulnerabilities, the corresponding weaknesses to each vulnerability ($CWE_{a_{i}}\left(t\right)$) are added, along with the corresponding attack patterns ($CAPEC_{w_{i}}\left(t\right)$) for each weakness. If there is no known vulnerability in an asset, then there will be no weaknesses. Meanwhile, it would be possible to have a known vulnerability in an asset, but no known weakness or attack pattern for that vulnerability. Finally, more than one CAPEC can be assigned to the same weakness. Consequently, it would be common to have a set of possible CAPECs that can be used to exploit the same weakness. It is worth noting that not all of them could be applied in every scenario.

Step 5—Computing Metrics and tracking the SUT. At this point, the EDG of the SUT is completed with all the public information that can be gathered. This last step is to calculate the metrics defined (for further information, see Section 3.2), generate the corresponding reports and track the state of the SUT for possible updates in the information of the model. This step is always triggered when the SUT is updated. This can imply that a new asset can appear, an old asset can disappear, an old vulnerability can be patched, or a new one can appear in the SUT. All of these scenarios will be reflected in the model as they arise during its life cycle.

### 3.2. Security Metrics

The EDG model that was proposed in the previous sections is by itself capable of representing the internal structure of the SUT, and it can display it graphically for the user. This representation not only includes the internal assets of the SUT, but also captures their relationships, existing vulnerabilities, and weaknesses. Moreover, assets, vulnerabilities, and weaknesses are easily identified using their corresponding CPE, CVE, and CWE values, respectively. Altogether, this constitutes a plethora of information that the model can use to improve the development and maintenance steps of the SUT, enhance its security, and track its status during its whole life cycle. Metrics are a great tool to integrate these features into the model.

Metrics can serve as a tool to manage security, make decisions, and compare results over time. They can also be used to systematically improve the security level of an industrial component or to predict its security level at a future point in time.

In this section, the basic definitions that serve as the foundation of the metrics are described. Then, the proposed metrics are introduced to complement the functionality of the EDG model. The main feature of these metrics is that they all depend on time as a variable, so it is possible to capture the actual state of the SUT, track its evolution over time, and compare the results.

#### 3.2.1. Basic Definitions

In this section, the basic concepts on which the definitions of the metrics will be based are formalized.

**Definition** **2.** *The set of all possible weaknesses at a time t is represented as CWE(t), where*

(1) $$CWE\left(t\right)=\left\{cwe_{1},\ldots ,cwe_{m}\right\}$$

*and m is the total number of weaknesses at time t. This set contains the whole CWE database defined by MITRE [38].*

**Definition** **3.** *The set of all of the possible vulnerabilities at a time t is represented as CVE(t) where*

(2) $$CVE\left(t\right)=\left\{cve_{1},\ldots ,cve_{p}\right\}$$

*and p is the total number of vulnerabilities. This set contains the whole CVE database defined by MITRE [34].*

**Definition** **4.** *The set of all possible attack patterns at a time t is represented as CAPEC(t), where*

(3) $$CAPEC\left(t\right)=\left\{capec_{1},\ldots ,capec_{q}\right\}$$

*and q is the total number of attack patterns at time t. This set contains the whole CAPEC database defined by MITRE [82].*

**Definition** **5.** *The set of weaknesses of an asset $a_{i}$ at a time t is defined as*

(4) $$\begin{array}{c} CWE_{a_{i}}\left(t\right)=\left\{cwe_{j}\right|cwe_{j}\;\mathrm{is}\;\mathrm{in}\;\mathrm{the}\;\mathrm{asset}\;a_{i}\;\mathrm{at}\;\mathrm{time}\;t\wedge cwe_{j}\in CWE\left(t\right)\\ \wedge \forall k\neq j,cwe_{j}\neq cwe_{k}\} \end{array}$$

*From this expression, the set of all the weaknesses of a particular asset throughout its life cycle is defined as*

(5) $$CWE_{a_{i}}=\overset{T}{\underset{t=1}{⋃}}CWE_{a_{i}}\left(t\right)$$

*where $|CWE_{a_{i}}|$ is the total number of non-repeated weaknesses in its entire life cycle.*

**Definition** **6.** *The set of vulnerabilities of an asset $a_{i}$ at a time t is defined as*

(6) $$CVE_{a_{i}}\left(t\right)=\left\{cve_{j}|cve_{j}\;is\;in\;the\;asset\;a_{i}\;at\;time\;t\wedge cve_{j}\in CVE\left(t\right)\right\}$$

*From this expression, the set of vulnerabilities of an asset throughout its entire life cycle is defined as*

(7) $$CVE_{a_{i}}=\overset{T}{\underset{t=1}{⋃}}CVE_{a_{i}}\left(t\right)$$

*where $|CVE_{a_{i}}|$ is the total number of vulnerabilities in its entire life cycle.*

**Definition** **7.** *The set of weaknesses of a SUT A with n assets at a time t is defined as:*

(8) $$CWE_{A}\left(t\right)=\overset{n}{\underset{i=1}{⋃}}CWE_{a_{i}}\left(t\right)$$

**Definition** **8.** *The set of vulnerabilities of a SUT A with n assets at a time t is defined as:*

(9) $$CVE_{A}\left(t\right)=\overset{n}{\underset{i=1}{⋃}}CVE_{a_{i}}\left(t\right)$$

**Definition** **9.** *The set of vulnerabilities associated with the weakness $cwe_{j}$ and to the asset $a_{i}$ at a time t is defined as:*

(10) $$CVE_{a_{i}|cwe_{j}}\left(t\right)=\left\{cve_{k}|cve_{k}\;\mathrm{associated}\;\mathrm{with}\;\mathrm{weakness}\;cwe_{j}\;\mathrm{and}\;\mathrm{to}\;\mathrm{asset}\;a_{i}\;\mathrm{at}\;\mathrm{time}\;t\right\}$$

It is worth noting that CWE is used as a classification mechanism that differentiates CVEs by the type of vulnerability that they represent. A vulnerability will usually have only one associated weakness, and weaknesses can have one or more associated vulnerabilities [85].

**Definition** **10.** *The partition j of an asset $a_{i}$ at time t conditioned by a weakness $cwe_{k}$ is defined as*

(11) $$CVE_{a_{i}|cwe_{k}}\left(t\right)=\left\{cwe_{l}|cwe_{l}=cwe_{k}\wedge cwe_{l}\in CVE_{a_{i}}\left(t\right)\right\}$$

**Definition** **11.** *The partition j of the SUT A at time t conditioned by a weakness $cwe_{k}$ is defined as*

(12) $$CVE_{A|cwe_{k}}\left(t\right)=\left\{cwe_{l}|cwe_{l}=cwe_{k}\wedge cwe_{l}\in CVE_{A}\left(t\right)\right\}$$

**Definition** **12.** *The set of attack patterns associated to a weakness $w_{i}$ at a time t is defined as*

(13) $$\begin{array}{c} CAPEC_{w_{i}}\left(t\right)=\left\{capec_{j}\right|capec_{j}\;\mathrm{can}\;\mathrm{exploit}\;\mathrm{weakness}\;w_{i}\;\mathrm{at}\;\mathrm{time}\;\\ t\wedge capec_{j}\in CAPEC\left(t\right)\} \end{array}$$

**Definition** **13.** *The set of metrics that are defined in this research work based on the EDG model is defined as*

(14) $$M=\{m_{1},\ldots ,m_{r}\}$$

*where r is the total number of metrics. This set can be extended, defining more metrics according to the nature of the SUT.*

#### 3.2.2. Metrics

This section will describe the metrics that were defined based on the EDG model and the previous definitions. Although it might seem trivial, the most interesting feature of these metrics is that they all depend on time. Using time as an input variable for the computation of the metrics opens the opportunity to track results over time, compare them, and analyze the evolution of the status of the SUT. Furthermore, some metrics take advantage of time to generate an accumulated value, giving information about the life cycle of the SUT. Table 2 shows all of the proposed metrics, their definition, and their reference values.

In addition to the metrics in Table 2, the model allows the definition of other types of metrics according to the analysis to be performed, and the nature of the SUT (e.g., the vulnerability evolution function for SUT *A* up to time *t* for all vulnerabilities could be defined as the linear regression of the total number of vulnerabilities in each time *t* for SUT *A*, or using any other statistical model).

### 3.3. Properties

Together, the EDG model and the defined metrics exhibit a series of characteristics that make them suitable for vulnerability assessment. These properties represent an advantage over the techniques reviewed in the state of the art, including automatic inference of root causes, spatial and temporal distribution of vulnerabilities, and prioritization of patching, which will be described in the following subsections.

#### 3.3.1. Automatic Inference of Root Causes

Each CWE natively contains information that is directly related to the root cause of a vulnerability. From this information, new requirements and test cases can be proposed.

#### 3.3.2. Spatial and Temporal Distribution of Vulnerabilities

The key feature of the proposed model is the addition of the temporal dimension in the analysis of vulnerabilities. This makes it possible to analyze the location of the vulnerabilities both in space (in which asset) and time (their recurrence), which allows us to track the state of the device throughout the whole life cycle. This approach also enables further analysis of the SUT, by updating data in the model, such as new vulnerabilities that are found or new patches that are released.

**Figure 6:**
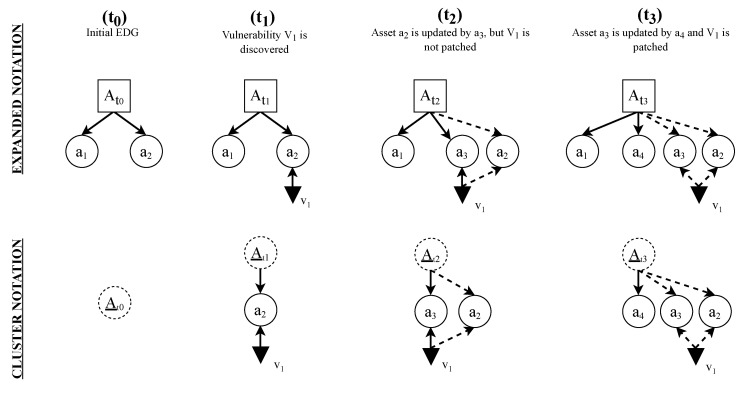
Representation of the temporal behavior in the graphical model using the two kinds of dependencies of the model. It is worth mentioning that these graphs could be further simplified by taking advantage of the cluster notation, as shown at the bottom of this figure.

Each time that a new vulnerability is found, or an asset is patched (i.e., via an update), the initial EDG is updated to reflect those changes. An example of this process can be seen in Figure 6.

At time $t_{0}$, the initial graph of the SUT *A* is depicted in Figure 6. Because there is no vulnerability at that time, this graph can be simplified using the cluster notation, with just a cluster containing all assets. At time $t_{1}$, a new vulnerability that affects the asset $a_{2}$ is discovered. At time $t_{2}$, the asset $a_{2}$ is updated. This action creates a new version of asset $a_{2}$, asset $a_{3}$. Because the vulnerability was not corrected in the new update, both versions contain the vulnerability that was initially presented in asset $a_{2}$. Finally, at time $t_{3}$, the asset $a_{3}$ is updated to its new version $a_{4}$, and the vulnerability is corrected.

This approach enables a further analysis of the SUT, including updated data, according to new vulnerabilities that are found or new patches that are released.

#### 3.3.3. Patching Policies Prioritization Support

The proposed model is not only able to include known vulnerabilities associated with an asset, but it also provides a relative importance sorting of vulnerabilities by CVSS. Relying on the resulting value, it is possible to assist in the vulnerability patching prioritization process. Furthermore, the presence of an existing exploit for a known vulnerability can be also be taken into account, when deciding which vulnerabilities need to be patched first. A high CVSS value combined with an available exploit for a given vulnerability is a priority when patching.

## 4. Real Use Case

In this section, we applied the EDG model to analyze the vulnerabilities of the OpenPLC project. For the sake of simplicity, the use case focuses on version one (V1) of OpenPLC. We centered the analysis on two of the assets that compose this version of the project: libssl and nodejs.

OpenPLC is the first functional standardized open-source Programmable Logic Controller (PLC), both in software and hardware [86,87,88,89]. It was mainly created for research purposes in the areas of industrial and home automation, the Internet of Things (IoT), and SCADA. Given that it is the only controller that provides its entire source code, it represents an engaging low-cost industrial solution—not only for academic research but also for real-world automation [90,91].

### 4.1. Structure of OpenPLC

The OpenPLC project consists of three parts:

1. Runtime: It is the software that plays the same role as the firmware in a traditional PLC. It executes the control program. The runtime can be installed in a variety of embedded platforms, such as the Raspberry Pi, and in Operating Systems (OSs) such as Windows or Linux.
2. Editor: An application that runs on a Windows or Linux OS that is used to write and compile the control programs that will be later executed by the runtime.
3. HMI Builder: This software is to create web-based animations that will reflect the state of the process, in the same manner as a traditional HMI.

When installed, the OpenPLC runtime executes a built-in webserver that allows OpenPLC to be configured and new programs for it to run to be uploaded. In this use case, we focused the analysis on the runtime of OpenPLC V1.

### 4.2. Setup through the Analysis

Ubuntu Linux was selected as the platform to install the runtime of OpenPLC V1. Ubuntu Linux provides comprehensive documentation, previous versions are accessible, and software dependencies can easily be obtained.

To make the analysis fair, a contemporary operating system was selected, according to the version of Ubuntu that was available at the release time of OpenPLC V1. The Long Term Support (LTS) version was chosen because industry tends to work with the most stable version available of any software and security updates are provided for a longer time. OpenPLC V1 was released in 2016/02/05, so we found that Ubuntu 14.04 LTS was the most suitable version [92]. The setup consisted of OpenPLC installed on 14.04 LTS Ubuntu Linux in a virtual machine. All configuration options were by default.

### 4.3. Building the EDG

**Figure 7:**
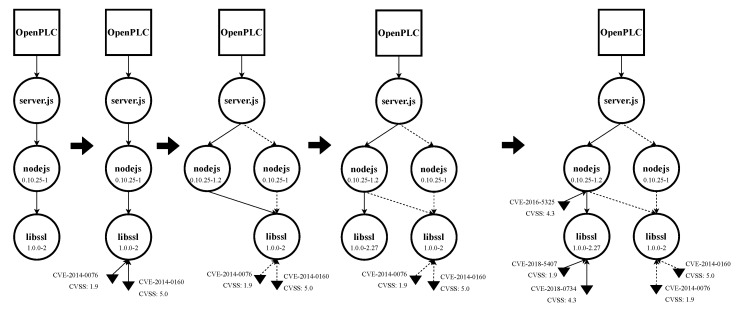
Temporal evolution of the EDG for OpenPLC V1 for both libss and nodejs.

**Figure 8:**
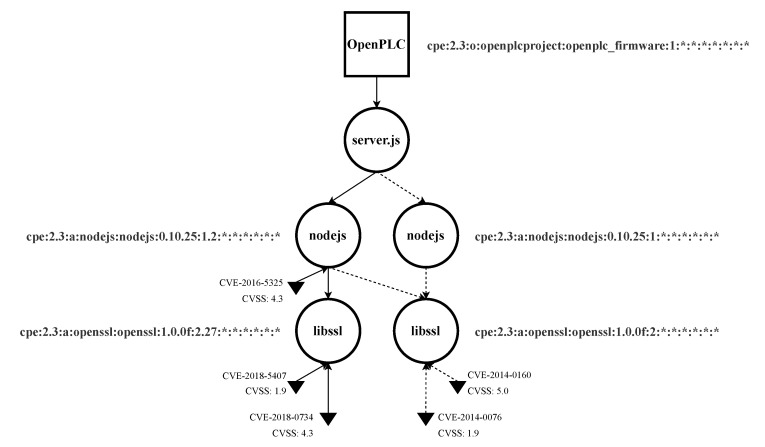
Final EDG for libssl and nodejs integrating all the updates for Ubuntu Linux 14.04 for amd64 architecture.

We built the entire EDG for OpenPLC V1, which can be found in Appendix B. Nevertheless, for the sake of clarity, we restricted this analysis in two ways: (1) focusing on two assets, libssl and nodejs; (2) integrating only security updates (discarding updates that introduced more functionalities). Table 3 shows the updates and their date of availability for both libssl [93] and nodejs [94] for Ubuntu 14.04 LTS. There were two security updates available for the amd64 architecture for each asset. Figure 7 illustrates step by step the partials EDG graphs, and Figure 8 shows the final EDG with all the updates merged in a single graph.

### 4.4. Analysis of the EDG

Using Figure 8 as reference, we can analyze the obtained EDG:

1 Analysis of the induced EDG model: The structure, assets, and dependencies are the focus of this first step.
  We can observe that libssl is used by nodejs, and they are not at the same level of the hierarchy. So vulnerabilities could propagate upwards through the EDG.
2 Vulnerability analysis: Vulnerability number, distribution, and severity are analyzed in this step. A proposal for vulnerability prioritization is also generated.
  We can highlight that nodejs had one vulnerability discovered after its first update, whereas libssl had vulnerabilities in both periods of time. We could argue that, as nodejs is the most accessible asset from the exterior, its vulnerabilities should be first addressed, even though the associated CVSS is not the highest one.
3 Weaknesses analysis: Finally, the root cause of each vulnerability is found. In this step, new requirements, test cases, and training activities are proposed based on the results of the analysis.
  Table 4 shows the root cause for each vulnerability. Using this data, new requirements, test cases, and training activities were proposed (see Appendix C).

## 5. Conclusions and Future Work

Vulnerability analysis is a critical task which ensures the security of industrial components. The EDG model that we propose performs continuous vulnerability assessment throughout the entire life cycle of industrial components. The model is built on a directed graph-based structure and a set of metrics based on globally accepted security standards. Metrics can be used by the model to improve the development process of the SUT, enhance its security, and track its status. The key feature of the proposed model is the addition of the temporal dimension in the analysis of vulnerabilities. The location of vulnerabilities can be analyzed in both space (in which asset) and time (their recurrence), which allows the state of the device to be tracked throughout the whole life cycle.

The model was successfully applied to the OpenPLC use case, which demonstrated its advantages, applicability, and potential. The use case showed that the model can assist in updating management activities, applying patching policies, launching training activities, and generating new test cases, and requirements. This has significant implications for cybersecurity evaluators, as it can serve as a starting point for identifying vulnerabilities, weaknesses, and attack patterns.

Further research will enhance the EDG by adding a mathematical model to aggregate the values of the CVSS metric for each asset, and a value for the whole SUT. This will enable the comparison of different SUTs over time. More improvements will be made in the prioritization of patching, taking into account the context and the functionalities of the SUT. Finally, historical information about the developers can be integrated into the EDG model to predict future vulnerabilities.

## Figures and Tables

**Table 1:** Overview of the information that is necessary to define each of the EDG elements.

Symbol	Notation	Meaning	Values
□	*A(t)*	Root Node/ Device Node	$CPE_{current}$
◯	a(t)	Asset Node	$CPE_{previous},CPE_{current},CWE_{a_{i}}\left(t\right)$
◌	$\underline{a}\left(t\right)$	Cluster	$\left\{CPE_{previous},CPE_{current},CWE_{a_{i}}\left(t\right)\right\},\left\{CVE_{a_{i}}\left(t\right),CVSS_{v_{i}}\left(t\right),CAPEC_{w_{i}}\left(t\right)\right\},\left\{Dependencies\right\}$
▾	v(t)	Known Vulnerability Node	$CVE_{a_{i}}\left(t\right),CVSS_{v_{i}}\left(t\right),CAPEC_{w_{i}}\left(t\right)$
⟶	e(t)	Dependency Relation	—
⤏	e(t)	Updated Asset/ Patched Vulnerability	—

**Table 2:** Proposed metrics for the model.

Metric		Definition	Reference Value
VULNERABILITIES	$M_{0}\left(A\right)=\frac{|CVE_{A}\left(t\right)|}{n(t)}$	Arithmetic mean of vulnerabilities in the SUT *A*, where n(t) is the number of assets in a SUT at a time *t*. $M_{0}$ shows how many vulnerabilities would be present in each asset if they were evenly distributed among the assets of the SUT. The result of $M_{0}$ can serve as a preliminary analysis of the SUT, related to the criticality of its state. From Equation (8).	$M_{0}<1$: The number of vulnerabilities is lower than the number of assets. $M_{0}\geq 1$: Every asset has at least one vulnerability.
	$M_{1}\left(A,t\right)=\left|CVE_{A}\left(t\right)\right|$	Number of vulnerabilities in a SUT *A* at time *t*. From Equation (8).	Ideally, the values of $M_{1}$ should be zero (no vulnerability in *A*), but the lower the value of $M_{1}$, the better.
	$M_{2}\left(A\right)=\sum_{t=1}^{T}\left|CVE_{A}\left(t\right)\right|=\sum_{t=1}^{T}M_{1}\left(A,t\right)$	Number of vulnerabilities in a SUT *A* throughout its entire life cycle *T*. This metric computes the accumulated value of the number of vulnerabilities of a SUT throughout its entire life cycle. From Equation (8).	The lower the value of $M_{2}$, the better.
	$M_{3}\left(a_{i},t\right)=\left|CVE_{a_{i}}\left(t\right)\right|$	Number of vulnerabilities in an asset $a_{k}$ at time *t* The values of $M_{3}$ can be useful during a vulnerability analysis, or when performing a penetration test, to identify the asset with more vulnerabilities. From Equation (6)	Ideally, the value of $M_{3}$ should be zero.
	$M_{4}\left(a_{k},t\right)=\frac{|CVE_{a_{k}}\left(t\right)|}{\sum_{i=1}^{n}\left|CVE_{a_{i}}\left(t\right)\right|}$	Relative frequency of vulnerabilities of the asset $a_{k}$ at a time *t*. From Equation (6).	Ideally, the value of $M_{4}$ should be zero, or at least $M_{4}$$\leq \frac{1}{n(t)}$, being n(t) the number of assets in the SUT. This value can also be expressed as the percentage of vulnerabilities of asset $a_{i}$ respect to the total number of vulnerabilities in the SUT, $M_{4}$$\left(a_{k},t\right)=\frac{|CVE_{a_{k}}\left(t\right)|}{\sum_{i=1}^{n}\left|CVE_{a_{i}}\left(t\right)\right|}\cdot 100$
	$M_{5}\left(a_{i},cwe_{j},t\right)=\left|CVE_{a_{i}|cwe_{j}}\left(t\right)\right|$	Multiplicity of weakness $cwe_{j}$ of the asset $a_{i}$ at a time *t*. This metric represents the number of times a weakness is present among the vulnerabilities of the asset $a_{i}$. This is possible because a vulnerability can have associated the same weakness as other vulnerabilities. From Equation (9).	Ideally, the value of $M_{5}$ should be zero, or at least, $M_{5}$$\leq \frac{|CVE_{A|cwe_{j}}\left(t\right)|}{n(t)}$, being n(t) the number of assets in the SUT. The value of the metric could be further narrowed by assuming that $cwe_{j}$ will be present in all but one asset, so $M_{5}$$\leq \frac{|CVE_{A|cwe_{j}}\left(t\right)|}{n(t)-1}$ to be in acceptable values.
	$M_{6}\left(A,cwe_{j},t\right)=\left|CVE_{A|cwe_{j}}\left(t\right)\right|$	Multiplicity of weakness $cwe_{j}$ of the SUT *A* at a time *t*. This metric represents the number of times a weakness is present among the vulnerabilities of the SUT *A*. From Equation (11).	Ideally, the value of $M_{6}$ should be zero.
WEAKNESSES	$M_{7}\left(A,t\right)=\left|CWE_{A}\left(t\right)\right|$	Number of weaknesses in a SUT *A* at time *t*. From Equation (7).	Ideally, the value of $M_{7}$ should be zero (no weakness in *A*), but the lower the value of $M_{7}$, the better.
	$M_{8}\left(A\right)=\sum_{t=1}^{T}\left|CWE_{A}\left(t\right)\right|=\sum_{t=1}^{T}M_{7}\left(A,t\right)$	Number of weaknesses in a SUT *A* throughout its entire life cycle *T*. This metric computes the accumulated value of weaknesses of a SUT throughout its entire life cycle. From Equation (7)	The lower the value of $M_{8}$, the better.

**Table 3:** Update information of both libssl and nodejs.

Asset	1st Update	Solved Vulnerabilities (CVSS)	2nd Update	Solved Vulnerabilities (CVSS)
libssl	2014/04/07	CVE-2014-0076 (1.9) CVE-2014-0160 (5.0)	2018/12/06	CVE-2018-5407 (1.9) CVE-2018-0734 (4.3)
nodejs	2014/03/27	—	2018/08/10	CVE-2016-5325 (4.3)

**Table 4:** Relationship between vulnerabilities and weaknesses for both libssl and nodejs.

CVE	CVSS	CWE	Description
CVE-2014-0076	1.9	CWE-310	Cryptographic Issues
CVE-2014-0160	7.5	CWE-119	Improper Restriction of Operations within the Bounds of a Memory Buffer
CVE-2016-5325	6.1	CWE-113	Improper Neutralization of CRLF Sequences in HTTP Headers (‘HTTP Response Splitting’)
CVE-2018-0734	5.9	CWE-327	Use of a Broken or Risky Cryptographic Algorithm
CVE-2018-5407	4.7	CWE-203 CWE-200	Observable Discrepancy Exposure of Sensitive Information to an Unauthorized Actor

**Figure A1:**
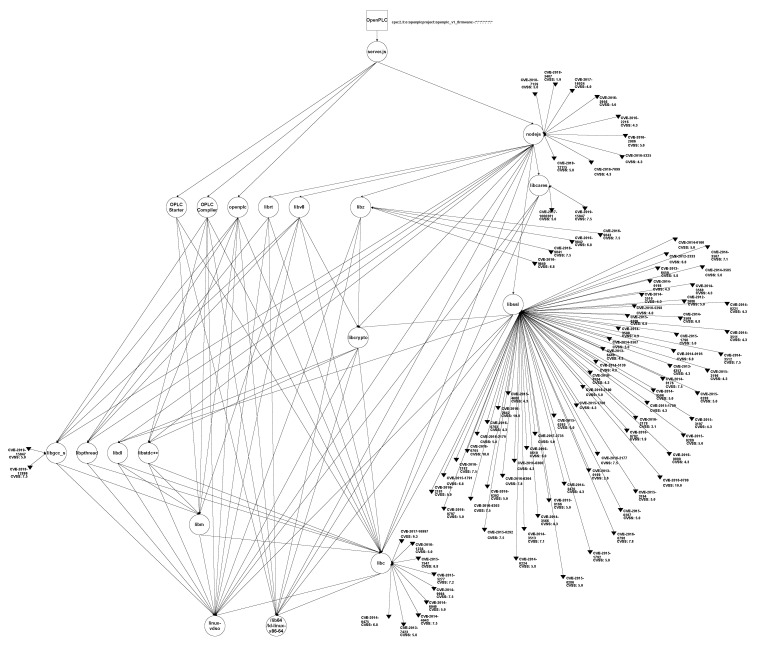
EDG for OpenPLC V1. Notice that, for simplicity, CWE and CAPEC values are omitted, and only the CPE identifier of the SUT is shown.

## Data Availability

Not applicable.

## Abbreviations

The following abbreviations are used in this manuscript:

AI	Artificial Intelligence
CC	Common Criteria
CAPEC	Common Attack Pattern Enumeration and Classification
COTS	Commercial Off-The-Shelf
CPE	Common Platform Enumeration
CPS	Cyber-Physical System
CVE	Common Vulnerabilities and Exposures
CVSS	Common Vulnerability Scoring System
CWE	Common Weakness Enumeration
EAL	Evaluation Assurance Level
EDG	Extended Dependency Graph
ES	Embedded System
IACS	Industrial Automation Control System
IoT	Internet Of Things
PLC	Programmable Logic Controller
SUT	System Under Test

## Appendix A. Applicability in the Context of ISA/IEC 62443

In this section, the potential application of the proposed EDG model to the existing security standards is described. The proposed EDG model can be used isolated by itself, or in combination with other techniques that complement the analysis. In this sense, the EDG model can be used to enhance some tasks in the security evolution processes defined by security standards.

The ISA/IEC 62443-4-1 standard specifies 47 process requirements for the secure development of products used in industrial automation and control systems [49]. Thus, the EDG model was developed to enhance the execution of one of those requirements defined by the standard: the “SVV-3: Vulnerability testing” requirement, serving as a support for the execution of Practice 5—Security Verification and Validation testing. According to the SVV-3 requirement, both known and unknown vulnerability analysis has to be performed. The EDG model proposed in this research work is intended to support the identification of known vulnerabilities, their dependencies, and the possible consequences of their propagation, yielding the opportunity to analyze them systematically. Nevertheless, more requirements of the ISA/IEC 62443 can be mapped to one or more of the metrics defined in this research work. Using this relationship, it is possible to apply the EDG model to enhance the analysis and review of the following requirements:

### Appendix A.1. Security Requirements—2: Threat Model (SR-2)

“A process shall be employed to ensure that all products have a threat model specific to the current development scope of the product. The threat model shall be reviewed and verified periodically” [49]. The proposed EDG model can serve as an abstraction of the threat model that has to be obtained. Moreover, the standard states that this threat model has to be reviewed periodically for updates. Given that the EDG of a given SUT evolves with every update, the threat model would be always up-to-date. Potential threats and their severity using the CVSS can also be analyzed with this proposal. Finally, these results can be used to enhance the risk assessment of the SUT.

### Appendix A.2. Security Management—13: Continuous Improvement (SM-13)

“A process shall be employed for continuously improving the secure development life cycle” [49]. The EDG model can be used to identify recurrent issues in the development of an industrial component, due to its ability to track the state of a SUT over time. Consider the scenario where a piece of code contains an unknown vulnerability. For example, this code can implement a communication protocol or the generation of a cryptographic key. If this piece of code is recurrently integrated into many types of devices, then when they are released to the market, the end-users can identify that vulnerability and report it to the product supplier. The EDG can reflect the presence of that vulnerability. If an EDG is done for each type of device, then this problem can be detected beforehand. Using the CWE, the root problem can be detected. With this information, new training and corrective actions can be proposed to avoid this issue.

### Appendix A.3. Specification of Security Requirements—5: Security Requirements Review (SR-5)

“A process shall be employed to ensure that security requirements are reviewed, updated, and approved” [49]. As before, taking advantage of the previous scenario, the information extracted from the generated EDG model can be used to propose new requirements or to update the existing requirements.

### Appendix A.4. Security Verification and Validation Testing—4: Penetration Testing (SVV-4)

“A process shall be employed to identify and characterize security-related issues via tests that focus on discovering and exploiting security vulnerabilities in the product” [49]. The EDG model facilitates the identification of possible entry points to the SUT when carrying out a penetration test. In addition, existing attack patterns (CAPEC) and weaknesses (CWE) can serve as a starting point to discover unknown vulnerabilities and exploits.

### Appendix A.5. Management of Security-Related Issues—3: Assessing Security-Related Issues (DM-3)

“A process shall be employed for analyzing security-related issues in the product” [49]. When a new vulnerability is detected, end-users will report it to the product suppliers. Then, the corresponding EDG model of that SUT will be updated to reflect that change. This information, in addition to that previously contained in the EDG, can be used to obtain the severity value of the discovered vulnerability using the CVSS. This also facilitates the identification of root causes, related security issues, or the impact.

**Table A1:** Mapping between the developed metrics and the requirements they refer in the ISA/IEC 62443. SR (Security Requirements), SM (Security Management), SVV (Security Validation and Verification), DM (Management of Security-Related Issues).

Metric	SR-2	SR-5	SM-13	SVV-4	DM-3
$M_{0}\left(A\right)=\frac{|CVE_{A}\left(t\right)|}{n(t)}$	■	■	■	■	■
$M_{1}\left(A,t\right)=\left|CVE_{A}\left(t\right)\right|$	■	■	■	■	■
$M_{2}\left(A\right)=\sum_{t=1}^{T}\left|CVE_{A}\left(t\right)\right|=\sum_{t=1}^{T}M_{1}\left(A,t\right)$	□	■	■	□	□
$M_{3}\left(A,t\right)=\left|CVE_{a_{i}}\left(t\right)\right|$	■	■	■	■	□
$M_{4}\left(a_{k},t\right)=\frac{|CVE_{a_{k}}\left(t\right)|}{\sum_{i=1}^{n}\left|CVE_{a_{i}}\left(t\right)\right|}$	□	■	■	□	□
$M_{5}\left(a_{i},cwe_{j},t\right)=\left|CVE_{a_{i}|cwe_{j}}\left(t\right)\right|$	■	■	■	■	□
$M_{6}\left(A,cwe_{j},t\right)=\left|CVE_{A|cwe_{j}}\left(t\right)\right|$	■	■	□	■	■
$M_{7}\left(A,t\right)=\left|CWE_{A}\left(t\right)\right|$	■	□	□	■	■
$M_{8}\left(A\right)={⋃}_{t=1}^{T}\left|CWE_{A}\left(t\right)\right|={⋃}_{t=1}^{T}M_{7}\left(A,t\right)$	□	■	■	□	□

Finally, the ISA/IEC 62443-4-2 document defines four types of components of an IACS (i.e., software applications, embedded devices, host devices, network devices) [95]. The proposed model is capable of representing the inherent complexity of each of them.

## Appendix B. EDG for OpenPLC V1

This appendix contains the generated EDG for OpenPLC V1.

## Appendix C. Proposed Requirements, Training, and Test Cases

In this appendix, we show the generated requirements, training, and test cases from the EDG model of OpenPLC V1.

**Table A2:** An example of generated requirements for OpenPLC V1.

CWE ID	Requirements
CWE-119	Use languages that perform their own memory management.
CWE-119	Use libraries or frameworks that make it easier to handle numbers without unexpected consequences. Examples include safe integer handling packages such as SafeInt (C++) or IntegerLib (C or C++).
CWE-119, CWE-200	Use a CPU and operating system that offers Data Execution Protection (NX) or its equivalent.
CWE-190, CWE-200	Ensure that all protocols are strictly defined, such that all out-of-bounds behaviors can be identified simply, and require strict conformance to the protocol.
CWE-310	Clearly specify which data or resources are valuable enough that they should be protected by encryption. Require that any transmission or storage of this data/resource should use well-vetted encryption algorithms. Up-to-date algorithms must be used, and the entropy of the keys must be sufficient for the application.
CWE-113	Use an input validation framework such as Struts or the OWASP ESAPI Validation API.
CWE-113	Assume all input is malicious. Use an "accept known good" input validation strategy, i.e., use a list of acceptable inputs that strictly conform to specifications. Reject any input that does not strictly conform to specifications, or transform it into something that does.
CWE-113	Hard-code the search path to a set of known-safe values (such as system directories), or only allow them to be specified by the administrator in a configuration file. Do not allow these settings to be modified by an external party.
CWE-119	Run or compile the software using features or extensions that automatically provide a protection mechanism that mitigates or eliminates buffer overflows.
CWE-119	Replace unbounded copy functions with analogous functions that support length arguments, such as strcpy with strncpy. Create these if they are not available.

**Table A3:** Example of proposed training for OpenPLC V1.

CWE ID	Training
CWE-113, CWE-119	Identification of all potentially relevant properties of an input (length, type of input, the full range of acceptable values, missing or extra inputs, syntax, consistency across related fields).
CWE-113, CWE-119	Input validation strategies.
CWE-113, CWE-119, CWE-200	Allowlists and Denylists.
CWE-113, CWE-119	Character encoding compatibility.
CWE-113, CWE-119	Buffer overflow detection during compilation (e.g., Microsoft Visual Studio /GS flag, Fedora/Red Hat FORTIFY_SOURCE GCC flag, StackGuard, and ProPolice).
CWE-113, CWE-119CWE-200	Secure functions, such as *strcpy* with *strncpy*. Create these if they are not available.
CWE-113, CWE-119CWE-190	Secure programming: memory management.
CWE-113, CWE-119	Understand the programming language’s underlying representation and how it interacts with numeric calculation.
CWE-113, CWE-119	System compartmentalization.
CWE-200, CWE-310	Certificate management.
CWE-200, CWE-310	Certificate pinning.
CWE-310	Encryption integration (do not develop custom or private cryptographic algorithms).
CWE-310	Secure up-to-date cryptographic algorithms.
CWE-200	Shared resource management.
CWE-200	Thread-safe functions.

**Table A4:** Example of generated test cases for OpenPLC V1.

Capec ID	Test Cases
CAPEC-119	Check for buffer overflows through manipulation of environment variables. This test leverages implicit trust often placed in environment variables.
CAPEC-119	Static analysis of the code: secure functions and buffer overflow.
CAPEC-119	Feed overly long input strings to the program in an attempt to overwhelm the filter (by causing a buffer overflow) and hoping that the filter does not fail securely (i.e. the user input is let into the system unfiltered)
CAPEC-119	This test uses symbolic links to cause buffer overflows. The evaluator can try to create or manipulate a symbolic link file such that its contents result in out-of-bounds data. When the target software processes the symbolic link file, it could potentially overflow internal buffers with insufficient bounds checking.
CAPEC-119	Static analysis of the code: secure functions and buffer overflow.
